# From experience to intention: how rural tourscape fosters destination sustainability through flow experience and memory

**DOI:** 10.3389/fpsyg.2026.1852245

**Published:** 2026-06-03

**Authors:** Haochen Xu, Ning Zhu, Jintao Zhu, Jinxiang Zhao, Changjiang Jin

**Affiliations:** 1 School of Architectural Art and Design, Luxun Academy of Fine Arts, Shenyang, China; 2 School of Fine Arts and Design, Hebei Normal University, Shijiazhuang, China; 3 Faculty of Education, Bangkokthonburi University, Bangkok, Thailand

**Keywords:** cognitive appraisal theory, flow experience, memory, revisit intention, rural tourscape, sustainable tourism development

## Abstract

**Introduction:**

In the context of increasingly high-pressure lifestyles, rural tourism has emerged as an important approach to restorative and meaningful travel and a key component of sustainable destination development. Based on Cognitive Appraisal Theory (CAT), this study develops a five-dimensional rural tourscape model, including natural environment, ambient atmosphere, hospitality culture, farming activities, and agricultural products, to examine their effects on tourists’ revisit intention.

**Methods:**

Data were collected from 443 valid questionnaires and analysed using Structural Equation Modelling (SEM) to examine the relationships among rural tourscape dimensions, flow experience, memory, and revisit intention. Fuzzy-set Qualitative Comparative Analysis (fsQCA) was further employed to complement the net-effect analysis and identify configurational pathways leading to high revisit intention.

**Results:**

The findings show that all five rural tourscape dimensions positively influence tourists’ flow experience. Flow experience directly enhances revisit intention and indirectly affects revisit intention through the formation of memorable experiences. The fsQCA results further reveal that no single tourscape dimension constitutes a necessary condition for high revisit intention; instead, multiple sufficient configurations exist.

**Discussion:**

By integrating SEM and fsQCA, this study provides a more comprehensive understanding of the psychological mechanisms underlying rural tourscapes and tourists’ revisit intention. The findings contribute to rural tourism research and offer practical implications for enhancing tourist experiences and promoting destination sustainability.

## 1 Introduction

In contemporary high-pressure societies, the pursuit of effective strategies for emotional regulation and mental well-being has become a pressing concern for both individuals and policymakers. It has been asserted that tourism functions as a valuable mechanism, in addition to leisure activities, to enhance psychological resilience and pro-social behaviors (Zhang et al., 2024; Zhu et al., 2025a,b). In this sense, rural tourism, which has its origins in natural scenery, local culture, and a more relaxed lifestyle, has become particularly significant in the post-pandemic period. In addition to providing opportunities for stress relief and the restoration of both the mind and body, rural tourism can be a direct component of global sustainability plans, particularly those focused on Good Health and Well-being (SDG 3) and Sustainable Cities and Communities (SDG 11) (Buhalis et al., 2023; Treviño-Villalobos et al., 2025).

In recent years, the research framework in tourism experiences has changed. Scholars have gradually shifted toward a more dynamic perspective of tourist satisfaction, considering contextual factors that influence tourists’ feelings and behavioral intentions (Hsu et al., 2021; Fan et al., 2024; Li J. et al., 2024; Li D. et al., 2024). As part of this change, researchers adopted the concept of experiencescape. This was later broadened into the idea of a tourscape, which aims to capture the full range of perceptions a tourist forms by engaging with a place’s nature, culture, and people (Liu and Lin, 2024). The tourscape framework has been widely applied in sustainable tourism research to explain tourists’ cognitive, affective, and conative responses, such as emotional arousal, destination identity, and revisit intention, particularly in eco-friendly contexts. Using the servicescape theory of Bitner (1992) and Zhang and Xu (2019) were the first to propose the concept of tourscape made up of four dimensions, namely natural, physical, social, and symbolic, and their role in enhancing emotional arousal, visitor loyalty, and destination identity was highlighted. Moreover, Torres-Moraga et al. (2024) suggested that the sustainability characteristics of tourscape, as a source of experiential stimuli, exert a significant influence on tourists’ construction of destination image, as well as their emotional experiences and behavioral intentions. In rural tourism, Liu and Lin (2024) localized the tourscape framework and subsequently proposed a concept called “rural tourscape.” They built a five-dimensional model encompassing (i) natural, (ii) ambient, (iii) hospitality, (iv) farming activities, and (v) agricultural products, demonstrating its impact on psychological restoration and place attachment. By analyzing user-generated content, Zhang and Omar (2024) identified four categories of rural tourism scenes (nature, food and accommodation, art, and activities) that elicit bodily, mental, and spiritual immersion, thereby enhancing participation and recommendation intention. Cai et al. (2026) investigated how rural tourism landscapes influence visitors’ environmental responsibility behaviors (ERB). The results indicate that elements such as atmosphere and agricultural products can directly affect visitors’ ERB, while other elements indirectly influence ERB by enhancing visitors’ sense of connectedness with nature and place attachment. The findings further confirm the role of rural tourism landscapes in promoting the sustainable development of destinations.

Despite the considerable amount of research that has investigated satisfaction, loyalty, revisit intention, and place attachment as outcomes, little is known about the impact of emotional experiences on revisit intention mediated by positive memory. This gap in understanding is particularly relevant in rural tourism, where a close experience with nature and local culture can leave a memorable experience that can be used as a catalyst for subsequent return behavior (Huang and Bu, 2022; Li J. et al., 2024; Li D. et al., 2024).

This paper helps fill this gap by presenting a holistic model explaining the psychological process linking rural tourscape factors, flow experience, and memory to revisit intention. The investigation is based on the principles of CAT; the research also includes flow experience and memory to evaluate how multidimensional experience factors trigger emotional feelings and convert them into long-lasting behavioral intentions. In order to give a more comprehensive picture, this paper combines SEM with fsQCA, which allows the identification of not only net effects but also several sufficient configurations of causality. Specifically, the research addresses four questions:

1 Do different types of rural tourscape elements significantly stimulate tourists’ flow experience?
2 Does flow experience affect revisit intention through memory?
3 Do flow experience and memory jointly mediate the relationship between rural tourscape and revisit intention?
4 What combinations of rural tourscape elements constitute sufficient configurations for high revisit intention from a configurational (fsQCA) perspective?

## 2 Literature review and hypothesis development

### 2.1 Cognitive appraisal theory

CAT, suggested by Lazarus (1984), assumes that two lines of evaluation occur upon exposure to external stimuli: the first is primary evaluation, identifying the relevance of circumstances to personal goals, and the second is secondary evaluation, assessing perceived capacity to handle the situation. Collectively, these appraisals determine the development and magnitude of emotional reactions. Emotional experiences, therefore, are not triggered directly by objective environments but by individuals’ cognitive interpretations of situational meaning (Meng and Luo, 2024).

The use of CAT in tourism studies has grown to elucidate how tourists’ perceptions of environmental quality, atmosphere, interactivity, and cultural proximity create pleasure and immersion, which in turn influence attitudes and behavioral intentions (Choi and Choi, 2019; Li J. et al., 2024; Li D. et al., 2024). The article by Ding and Hung (2021) also established that tourists’ perceptions of the destination atmosphere not only impact the level of immersion but also the richness and longevity of tourist memory. Perceived safety, cultural congruence, and emotional bonding seem to be multidimensional experiential characteristics appreciated by tourists in rural tourism (Liu and Lin, 2024; Song et al., 2025). In case such appraisals are found to be positive and controllable, positive psychological consequences are created, such as flow experience, memorable tourism episodes, and revisit intention (Zhang et al., 2022; Li and He, 2023).

Compared to the traditional S-O-R model, CAT provides a more precise causal explanation for stimuli from diverse sources, including emotions and behaviors. In modern tourism research, where the researcher aims at unraveling confusing phenomena like deep experiences and emotional attachments, CAT can overcome the constraints of the S-O-R model in an attempt to clarify ambiguity in stimulus intensity and behavioral actions. Immersion, memory, and behavioral intentions among tourists can be better studied through CAT. On these grounds, this research paper will follow a framework based on CAT to examine the impact of the dimensions of rural tourscape on revisit intention via flow experience and memory as mediators of their effects. The ensuing model explicates the cognitive-emotional processes of tourist decision-making and gives the theoretical basis for formulating sustainable destination management strategies.

### 2.2 Rural tourscape

The evolution of the ideas of servicescape, experiencescape, and tourscape reflect the academic community’s efforts to better understand how environments impact tourists. This lineage can be traced back to scene theory, an urban sociological approach, which considers a scene as an environment that is produced culturally by people, physical surroundings, and common values (Wu, 2014). Developed on this base, the concept was brought into service management in the form of the servicescape model, which is concerned with the influence of physical and atmospheric features on the way consumers perceive and act (Bitner, 1992). Experiencescape also expanded this view in the tourism literature by adding symbolic, sensual, and emotional connotations in place (O’Dell, 2005). In the same breath, tourscape expands on these advancements and offers a more holistic approach to tourism environments, a synthesis of natural, social, and cultural elements, which theorists can use to create a holistic understanding of the interactions between tourists and their environments. Table 1 provides a comparative overview of key studies within this conceptual family, highlighting the major dimensions associated with each framework.

**Table 1:** Summary of tourscape-related frameworks.

Study	Conceptual framework	Study context	Key dimensions
Bitner (1992)	Servicescape	Service management	Spatial settings, atmosphere, social interaction
O’Dell (2005)	Experiencescape	Tourism	Sensory, symbolic, emotional
Zhang and Xu (2019)	Tourscape	World heritage cultural-historic town	Natural, physical, social, symbolic
Liu and Lin (2024)	Rural tourscape	Rural tourism	Natural, ambient, hospitality, farming activities, agricultural products

In the context of rural tourism research, Liu and Lin (2024), building on the concepts of “servicescapes” and “experiencescenes,” proposed the notion of the “rural tourscape.” This concept builds on defining features of rural tourism, such as everyday life, social interaction, and cultural locality. It extends the application of experience-scene theory to rural settings. With the aid of this framework, the development of the study’s theoretical model is based on five dimensions, namely natural, ambient, hospitality, farming activities, and agricultural products. It aims to provide a holistic portrayal of the interaction process between tourists and rural areas. In particular, the natural dimension includes pastoral landscapes and ecoscapes, which provide the basic space for visual enjoyment and psychological relief. The ambient dimension is present in the tranquil pace of life and rich social configurations that can create emotional resonance and affection within tourists (Wang and Bai, 2020). The hospitality dimension reflects the warmth and friendliness of local residents, which generates a sense of belonging. Farming activities, such as fruit picking, cultivating, and cooking, encourage participation in rural production and daily life, thereby enhancing cultural identity. Finally, agricultural products, including local specialties and handicrafts, foster emotional satisfaction, memory formation, and behavioral loyalty (Liu and Lin, 2024). Collectively, these five dimensions constitute the core experiential elements of rural tourism. By stimulating immersion, fostering emotional connections, and reinforcing memorable experiences, the rural tourscape plays a vital role in strengthening tourists’ intention to revisit.

### 2.3 Rural tourscape and flow experience

Flow theory, initially proposed by Csikszentmihalyi (2000), defines flow as a psychological state of intense absorption, characterized by enjoyment and altered perception of time. The flow experience has been extensively used in tourism studies as one of the key indicators of immersion, subjective well-being, and satisfaction with tourism experiences (de Matos et al., 2021; Zhang et al., 2021; Tao et al., 2022; Liu et al., 2024).

Flow experience is considered to be dependent on tourists’ perception of the scene, with congruency, interactivity, and perceived control as significant antecedents (Tan et al., 2023). Ding and Hung (2021) relying on CAT, state that the quality of the experience, characterized by the atmosphere, cultural reaction, and contact with visitors, significantly enhances flow experience. The existing literature has also proven that flow is an emotional mediator between tourism experiences and memory, and further behavioral intentions. Consistent with the emerging wave of interest in sustainable tourism, researchers have highlighted the theoretical and practical significance of flow in rural settings, in which natural environments, richness of local culture, and opportunities to engage precondition positive prospects of immersive experiences (Lin et al., 2024).

A well-designed tourism scene has been shown to elicit flow states, evoke positive emotions, and strengthen travel-related memories, thereby enhancing revisit intention and behavioral loyalty (Wöran and Arnberger, 2012). In rural tourism, natural environments not only offer visual and sensory satisfaction but also provide psychological conditions conducive to flow (Carneiro et al., 2015). Low-disturbance, aesthetically pleasing environments have been identified as particularly favorable for the emergence of flow experience (Wanzer et al., 2020). Likewise, Hung and Chang (2024) highlight that rural natural surroundings reduce external distractions and foster calmness, thereby enhancing immersion. Taken together, prior research suggests that multidimensional elements of the rural tourscape can significantly stimulate flow experience, which subsequently fosters memory formation and revisit intentions (Seresinhe et al., 2019). Based on this rationale, this study proposes the following hypotheses:

*H1*. The natural dimension of rural tourscape has a significant positive effect on tourists’ flow experience.

In tourism experience studies, environmental atmosphere is an important external element impacting tourist emotion and thus subsequent immersive experiences (Liu et al., 2023; Weng et al., 2023). Prior research has shown that experience-related elements such as lighting, sound, crowd density, sense of order, and rhythm of interaction collectively constitute the servicescape, which shapes emotional responses, deepens immersion, and strengthens behavioral outcomes (Xu et al., 2025).

Within rural tourism contexts, a calm, pleasant, and relaxed atmosphere enhances tourists’ psychological comfort and perceived environmental congruence, thereby creating favorable conditions for the emergence of flow experience (Zhu et al., 2025a, 2025b). As Li J. et al. (2024); Li D. et al. (2024) note, flow experience represents a state of complete absorption in an activity, typically facilitated by a slower pace of life, natural social interactions, and a harmonious social atmosphere. Rural destinations often embody these features through “slow tourism” atmospheres characterized by closeness to nature, minimal external disturbance, and frequent face-to-face encounters (Zhai et al., 2023). These conditions collectively help tourists relax emotionally, focus attention, and achieve deep immersion (Zhang and Omar, 2024). Based on this, the following hypothesis is proposed:

*H2*. The ambient dimension of rural tourscape has a significant positive effect on tourists’ flow experience.

In the context of tourism experiences, hospitality, as a key social element in rural tourism, can significantly enhance tourists’ immersion and emotional engagement through genuine interactions and cultural identification between visitors and local residents or service providers (Christou and Sharpley, 2019). Displays of enthusiasm, respect, proactive service, and esteem by hosts not only enhance tourists’ emotional security and sense of cultural belonging, but also motivate engagement and emotional experiences (Chen et al., 2022).

Findings of studies in the field of festival tourism also point to hospitality as a significant antecedent of flow, enhancing tourists’ feelings of control over the environment and potential for self-expression, which consequently leads to a greater level of immersion (Williams, 2006). In the rural tourism environment, the perception of human warmth and the very localized nature of social interaction fosters a hospitality culture, which is a key source of emotional resonance and identity for tourists (Zhou and Wang, 2024). Thus, this study hypothesizes that:

*H3*. The hospitality dimension of rural tourscape has a significant positive effect on tourists’ flow experience.

Farming activities, as one of the major aspects of rural tourism participation, accelerate situational involvement and psychological absorption by means of tourists’ active participation and practical experience (Li J. et al., 2024; Li D. et al., 2024). The flow theory can give a good understanding of this phenomenon: flow states arise when the skills of an individual match the level of difficulty of an activity well (Shernoff and Csikszentmihalyi, 2009). Culturally embedded farming activities like planting, picking fruits, cooking, crafting, etc., may help tourists to be more focused, independent, and emotionally engaged, which results in a higher probability of entering a state of flow (de Matos et al., 2021).

Participatory activities are characterized by clear objectives and immediate feedback, giving tourists a sense of control and self-expression, key antecedents of flow (Li J. et al., 2024; Li D. et al., 2024). Highly participatory rural activities are more effective in creating deep immersion, creating long-lasting emotional memories, and strengthening behavioral intentions in comparison with traditional tourism models, as they require tourists to mainly observe rather than participate (e.g., sightseeing or performance watching) (Chi and Han, 2021; Zhai et al., 2023). Therefore, the following hypotheses are proposed:

*H4*. The farming activities dimension of rural tourscape has a significant positive effect on tourists’ flow experience.

As a cultural extension of rural tourism and a vehicle of place identity, creative agricultural products not only meet tourists’ functional consumption needs but also evoke emotional resonance and immersive experiences through their locality and uniqueness (Vujko et al., 2025). As tourists are exposed to regional specialties or intangible cultural heritage art that merges local culture, traditional methods, and lived experiences, they tend to reach a sense of place and contextual resonance that enhances the significance of the tourism experience (Tan et al., 2018).

Creative agricultural products also merge practicality and aesthetic value. Tourists are provided with explicit objectives, cultural newness, emotional gratification, and psychological states that are supportive of the development of flow experience (Liu and Zhao, 2024), whether in buying them, engaging in their production, or understanding their cultural point. In the setting of rural tourism, where these products have various local connotations and authenticity in their handicraft, they serve as tangible carriers of memorable experiences. They create emotional bonds, improve the cultural sense, and augment attachment to the destination, which boosts revisit intentions (Chi and Han, 2021). Based on this, involvement in agricultural products, which are rich in culture and participatory, is believed to be one of the main triggers of tourists’ immersion and flow experience. Based on this reasoning, the following hypothesis is proposed:

*H5*. The agricultural products dimension of rural tourscape has a significant positive effect on tourists’ flow experience.

### 2.4 Flow experience and memory

Tourism memory, a fundamental outcome variable assessing the depth and emotional value of tourist experiences, has become an important subject for understanding the psychological processes underlying behavioral reactions and destination loyalty (Kim, 2018). As well as being an individualized reenactment of travelling, memory manifests as a psychological basis involved in generating individual meaning, measuring experiential value, and developing place attachment (Tsai, 2016). In the experience economy, factors that influence the development of tourism memory include involvement, emotional arousal, and cognitive evaluation (Blumenthal and Jensen, 2019). The more tourists engage and interact directly with the environment and the stronger emotions they form, the more vivid, meaningful, and emotionally salient their memories (Wood, 2020).

This is where tourists are more sensitive to the situation and to themselves in their deep focus, enjoyment, and distortion of time. This increased sensitivity aids them in generating memories that are well organized and emotional (Baumann and Scheffer, 2010; Zhang et al., 2014). In countryside tourism, flow experiences are based on natural sceneries, cultural ambience, and participative experience, which help tourists to immerse themselves in the present to foster the creation of coherent and long-term memories (Carneiro et al., 2015).

These positive memories are easily recalled and can be repeatedly elicited in day-to-day life, reinforcing emotional bonding to the destination, enhancing destination image, and ultimately raising revisit intention (Kim and Youn, 2017). Thereby, tourism memory is not only a continuation of the experience but also a mediator that is decisive enough to transfer flow into long-term behavioral intentions. Based on this reasoning, the following hypotheses are proposed:

*H6*. Tourists’ flow experience has a significant positive effect on their tourism memory.

### 2.5 Memory and revisit intention

Revisit intention is a psychological characteristic or behavioral disposition of a person who visits a specific tourism destination once the experience is over (Kim and Youn, 2017). Being one of the fundamental concepts used to study tourist behavior, revisit intention indicates not only satisfaction and identification with the destination, but also psychological commitment to destination brand loyalty (Alegre and Cladera, 2009; Wang et al., 2024). Due to its capacity to be a good indicator of real revisit behavior, revisit intention has become a significant point in destination management and marketing plans, and has immediate impacts on both economic and long-term sustainability (Tan et al., 2023).

Tourism memory has been reputed to form a significant psychological foundation of revisit intention (Kim et al., 2022). Interestingly, Agapito et al. (2017) argue that it is at the end of travel that tourists construct memories; perceptual and emotional responses are incorporated and hence determine future actions. Positive memories that are related to feelings of pleasure, relaxation, and meaningfulness do not just reinforce affective attachment to destinations but are also often re-evoked in everyday life (Tung et al., 2017). Empirical data also show that the clarity of recall, emotional coloring, and frequency of recall are positively related to repeat visitation probability (Kim et al., 2022). This means that highly personal and emotionally loaded memories created during travel have a significant effect on revisit intention (Zhang et al., 2018). Thus, this study hypothesizes that:

*H7*. Tourism memory has a significant positive effect on tourists’ revisit intention.

### 2.6 Flow experience and revisit intention

Previous studies have highlighted that the flow experience that tourists achieve during travelling has a significant impact on their subsequent behavioral intentions. Such flow experience contributes to their revisit intention (Ding and Hung, 2021). The strong engagement and emotional fulfillment of tourists to a destination may produce strong feelings of affection and self-fulfillment, which create enhanced affection for the destination and additionally self-identification with the destination, psychologically (Tian et al., 2020). This has been orchestrated to ensure that tourists have a better overall assessment of the travel experience, as well as improving the likelihood of revisiting the same destination in the future as a result of enhanced flow experience (de Matos et al., 2021). Moreover, highly engaging experiences are more likely to be encoded in memory and emotional systems, providing emotional support for future revisit decisions (McGaugh, 2018). Therefore, it can be stated that flow experience is a process that enhances cognitive and emotional memory and significantly facilitates the formation of revisit intention. In summary, this paper proposes the following hypotheses:

*H8*. Tourists’ flow experience has a significant positive effect on their revisit intention.

**Figure 1:**
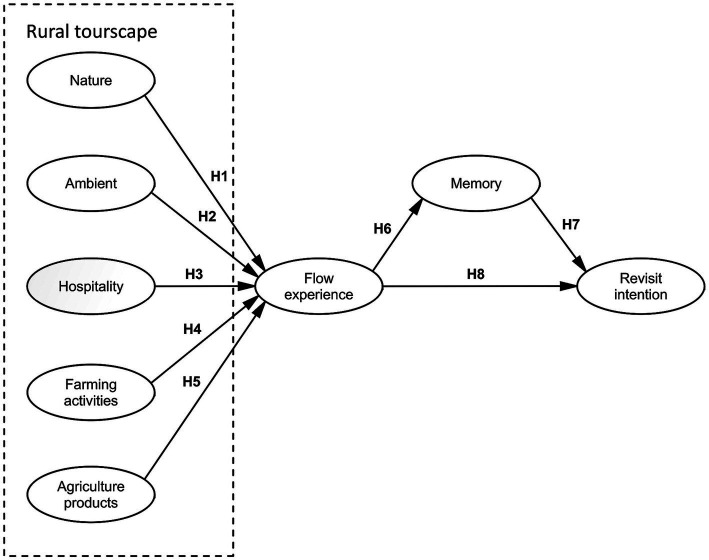
Conceptual model.

Grounded on the above research and assumptions, a conceptual model is presented in Figure 1.

## 3 Research methods

### 3.1 Research background and study sites

Qingdao’s coastal tourism plays an important role in economic development. In 2024, the city received approximately 140 million visitors and generated over 210 billion RMB in tourism revenue, demonstrating the strong growth of its coastal tourism sector (Qingdao Municipal People’s Government, 2025). In particular, Qingdao’s coastal fishing villages are regarded as key areas for coastal tourism development in China, contributing to economic growth, industrial transformation, rural revitalization, and cultural heritage preservation (Wang and Zhou, 2025; Hu et al., 2023). This study selected three typical coastal rural tourism destinations in Qingdao, Shandong Province—Qingshan Fishing Village, Gangdong Village, and Diaolongzui Village—as the field of interest in carrying out the study. These villages lie in the geographic conditions of the Laoshan Scenic Area, which is coastal and mountainous with abundant ecological resources and a rich cultural background. These types of places have become increasingly popular in recent years with the further development of rural revival and tourism development, becoming composite rural tourism destinations with particular emphasis on fishery culture experiences, coastal leisure, and homestay-based vacations.

### 3.2 Survey procedure and ethical considerations

The survey conducted using a questionnaire was focused on tourists who went to the above-mentioned villages in 2024. Convenience sampling was used, where tourists within the key tourism spots in the villages, including camping areas, food streets, and homestay areas, were invited to fill out the questionnaire voluntarily. The respondents were all adults aged 18 years and older. The questionnaire took approximately 15–20 min to complete. Participants accessed the questionnaire by scanning a QR code. The system offered an elaborate description of the purpose of the study, the topics, and the further use of data after the scan, as well as privacy protection. Participants could then proceed to the questionnaire after being fully informed and giving consent. On the ethical issues related to data collection, the questionnaire in this research explicitly indicates on the first page that all information will be used in academic research and is not concerned with any commercial operations. The respondents may drop out whenever they are not comfortable with the process of completion.

All methods followed the guidelines and regulations of the 1964 Helsinki Declaration and its later amendments. Since this study does not involve intervention and is low-risk, ethical review and approval were waived according to the institutional review boards at the School of Architectural Art and Design at Luxun Academy of Fine Arts. The participants provided their electronic informed consent to participate in this study.

To evaluate the intelligibility and stability of the questionnaire, a pilot study was conducted first, and then the official survey was initiated. In total, 443 valid responses were gathered. As stated in Weston and Gore (2006), SEM requires at least five times the number of parameters that are being estimated. The sample size of 443 valid responses is sufficient for SEM analysis, meeting the minimum requirement. The indirect effects were then tested through the bootstrapping method with 5,000 resamples, and a bias-corrected 95 percent confidence interval was employed to test whether the mediation paths were statistically significant.

### 3.3 Scales and measuring tools

According to the comprehensive overview of the existing sources and field research, a constructive questionnaire was designed independently and included five essential segments: (1) rural tourscape, (2) flow experience, (3) memory, (4) revisit intention, and (5) respondent demographic information.

The former part was designed according to the rural tourscape dimension scale introduced by Liu and Lin (2024), and it has 20 measures. The second part was based on a flow experience scale created by Chen et al. (2017), which was narrowed down to four main items. The third part included three memory items that were suggested by Ding and Hung (2021). The fourth section was that of revisit intention, assessing four items based on the framework of Torres-Moraga et al. (2024). The fifth section gathered demographic information of the respondents, such as gender, age, and education level.

To ensure the appropriateness of the scales in terms of language and cultural context, all measurement items were localized and adjusted according to the actual research setting. To evaluate subjective perceptions and attitudinal orientations, all items were measured using a five-point Likert scale (1 = “strongly disagree,” 5 = “strongly agree”).

In translating the original English scales into simplified Chinese, two master’s students who were conversant with the English language translated the English scales individually into Chinese. The research team analysed and combined the translations into a single one. Back-translations into English were performed by two other bilingual graduate students who had not been exposed to the original scales. The research team considered the back-translations and asked specialists in the field of translation and tourism to compose a review panel to estimate the consistency of the content.

A pilot test was held from July 6 to July 21, 2024, before the commencement of the formal survey in Qingshan Fishing Village, Gangdong Village, and Diaolongzui Village. Convenience sampling was used to distribute 100 questionnaires, and 81 valid responses were obtained, giving a response rate of 81%. The reliability analysis revealed that the coefficient of Cronbach’s alpha of all the scales ranged from 0.71 to 0.89, which means high internal consistency. Therefore, all 31 items were retained for the subsequent formal data collection.

### 3.4 Data collection and analysis

The formal data collection was conducted in Yumingsui Village from July 26 to August 31, 2024. A total of 500 tourists were invited by the research team to participate in the questionnaire survey. To ensure data quality, systematic screening for missing values and outliers was performed during the preprocessing stage. Specifically, any questionnaires completed in less than 180 s, as well as responses containing significant outliers, were excluded. After data cleaning, 443 valid questionnaires were retained, providing a solid foundation for subsequent empirical analysis. To address potential common-method bias, Harman’s single-factor test was conducted. The results showed that the first unrotated factor accounted for less than 40% of the total variance, indicating that common-method variance was not a serious concern.

Sample characteristics showed that respondents were primarily aged 18 and 60. Female participants accounted for 54.63%, slightly higher than the proportion of males (45.37%). In terms of education level, 68.62% of respondents had obtained an associate or bachelor’s degree, while 4.74% held a graduate degree (master’s or above). Details are presented in Table 2.

**Table 2:** Research participants’ general characteristics.

Variable	Category	Number	%
Gender	Female	242	54.63
	Male	201	45.37
Age (years)	18–30	216	48.76
	31–40	169	38.15
	41–60	46	10.38
	Over 60	12	2.71
Education	Junior high school or below	36	8.13
	High school or technical secondary school	82	18.51
	Associate or bachelor’s degree	304	68.62
	Graduate degree (Master’s or above)	21	4.74

1 CNY equals approximately 0.14 USD.

Data analysis was conducted using SPSS 27.0 and AMOS 26.0. The validation of the measurement model followed a two-step approach. First, confirmatory factor analysis (CFA) was employed to assess the convergent and discriminant validity of the scales. Second, Cronbach’s alpha coefficients were calculated for each subscale to examine internal consistency. SEM was used to test the overall path model and further analyze the direct, indirect, and total effects among variables.

Model fit was evaluated using several indices, including χ^2^/df, CFI, TLI, IFI, and RMSEA. A model was considered to have a good fit when χ^2^/df < 5.0, CFI, TLI, and IFI ≥ 0.90, and RMSEA ≤ 0.08, indicating that the proposed model demonstrated satisfactory goodness of fit (Schermelleh-Engel et al., 2003).

### 3.5 Additional configurational analysis: fsQCA

In addition to the symmetric and net-effect vision of SEM, this study also resorted to the use of fsQCA to define several adequate configurations that result in high revisit intention (Ragin, 2009; Fiss, 2011). The analysis was performed in accordance with the traditional fsQCA protocol: (1) the construction of composite indicators of the latent variables and direct calibration, which yielded fuzzy-set membership scores; (2) necessity analysis, which investigated whether any of the tourscape dimensions was a necessary condition of high revisit intention; and (3) sufficiency analysis involving the operations of truth tables and minimizing algorithms to identify causal configurations.

## 4 Result

Before conducting SEM, this study tested the normality of the sample data. According to the criteria proposed by Kim and Fesenmaier (2015), if the absolute value of skewness is less than 3 and the absolute value of kurtosis is less than 10, the data can be considered to approximately follow a normal distribution. The analysis showed that the skewness values of all measurement items ranged from −1.623 to −0.097, and the kurtosis values ranged from −0.540 to 3.187, all within acceptable limits. Hence, all variables satisfied the normality assumption and were deemed suitable for SEM analysis.

In addition, multicollinearity among the independent variables was assessed through linear regression analysis. The variance inflation factor values ranged from 1.168 to 2.058, well below the commonly accepted threshold of 10, while the tolerance values ranged from 0.486 to 0.856, all above the critical value of 0.1. According to the criteria proposed by Thompson et al. (2017), these results indicate that multicollinearity is not a serious concern in this study.

### 4.1 Measurement model

The reliability and validity of the measurement model were evaluated using the following indicators: standardized factor loadings, Cronbach’s alpha coefficients, composite reliability (CR), average variance extracted (AVE), and discriminant validity. According to Schumacker and Lomax (2004), standardized factor loadings should not be lower than 0.50. In this study, all item loadings ranged from 0.734 to 0.910, indicating strong explanatory power for each indicator, and all items were retained.

The Cronbach’s alpha coefficients for the five latent constructs ranged from 0.829 to 0.930 (see Table 2), all exceeding the 0.80 threshold, suggesting good internal consistency. In accordance with the criteria proposed by Fornell and Larcker (1981), CR values should be no less than 0.70, and AVE values should exceed 0.50. The CR and AVE values for all five constructs met these criteria (see Table 3), demonstrating acceptable convergent validity.

**Table 3:** Analysis results of construct validity and reliability.

Constructs/variables		Mean	Std.dev	Factor loadings	α	CR	AVE
Nature		3.300	0.850		0.930	0.930	0.728
NA1	The pastoral scenery here is truly charming.			0.841			
NA2	There is a wide variety of natural elements here, serving diverse functions.			0.863			
NA3	The integration of natural and cultural landscapes here is very harmonious.			0.877			
NA4	This place gives me a strong feeling of escaping from urban noise.			0.805			
NA5	The natural scenery here met or even exceeded my expectations.			0.878			
Ambient		3.156	0.797		0.893	0.895	0.682
AM1	The leisure atmosphere here is rich and appealing.			0.734			
AM2	The overall ambiance here is relaxed and pleasant.			0.827			
AM3	There is a good atmosphere for social interaction here.			0.891			
AM4	The pace of life here is very laid-back.			0.844			
Hospitality		3.237	0.763		0.907	0.908	0.711
HO1	The service staff here are warm and hospitable.			0.843			
HO2	The service staff here are attentive and thoughtful.			0.859			
HO3	The service staff here are kind and friendly.			0.806			
HO4	The service made me feel deeply cared for and connected to the local culture.			0.864			
Farming activities		3.841	0.824		0.910	0.913	0.725
FA1	Visitors can participate in the processing of agricultural or handicraft products.			0.910			
FA2	Visitors can engage in nature-based experiences like fishing or catching.			0.813			
FA3	Agricultural labor experiences, such as planting and harvesting, are available.			0.781			
FA4	Visitors can enjoy local specialty farmhouse cuisine.			0.895			
Agricultural products		3.794	1.091		0.897	0.898	0.745
AP1	There are many shops selling local specialty products.			0.855			
AP2	The local products here are rich in regional cultural characteristics.			0.892			
AP3	The handicrafts here reflect a strong sense of rural culture.			0.842			
Flow experience		3.671	1.066		0.910	0.910	0.716
FE1	I felt completely immersed during the rural tourism experience.			0.867			
FE2	I was fully focused and highly attentive during the rural tourism activities.			0.857			
FE3	Time seemed to fly by during my rural tourism experience.			0.826			
FE4	I was often in an optimal state during my rural tourism experience.			0.833			
Memory		3.676	0.962		0.829	0.838	0.622
ME1	I will have beautiful memories of this rural tourism experience.			0.791			
ME2	I will remember many positive aspects of this rural tourism experience.			0.751			
ME3	This rural tourism experience is truly unforgettable for me.			0.823			
Revisit intention		3.657	1.119		0.913	0.914	0.726
RI1	I would like to visit this place frequently.			0.818			
RI2	I really want to come back here again.			0.857			
RI3	I will always consider this destination as my first choice.			0.854			
RI4	It is very likely that I will revisit this destination.			0.878			

NA, nature; AM, ambient; HO, hospitality; FA, farming activities; AP, agricultural products; FE, flow experience; ME, memory; RI, revisit intention.

Furthermore, the square roots of AVE for each construct were greater than the inter-construct correlation coefficients, indicating satisfactory discriminant validity (see Table 4).

**Table 4:** Discriminate validity of the research model.

Constructs	NA	AM	HO	FA	AP	FE	ME	RI
NA	0.853							
AM	0.287	0.826						
HO	0.409	0.401	0.843					
FA	0.571	0.293	0.401	0.851				
AP	0.264	0.198	0.300	0.271	0.863			
FE	0.567	0.388	0.453	0.586	0.310	0.846		
ME	0.360	0.347	0.402	0.368	0.265	0.497	0.789	
RI	0.294	0.247	0.256	0.364	0.203	0.495	0.488	0.852

NA, nature; AM, ambient; HO, hospitality; FA, farming activities; AP, agricultural products; FE, flow experience; ME, memory; RI, revisit intention.

Regarding the structural model fit, the results indicated a good overall fit according to the criteria proposed by Hair et al. (2009). The model fit indices were as follows: χ^2^/df = 1.407, Tucker-Lewis Index (TLI) = 0.981, Comparative Fit Index (CFI) = 0.984, Incremental Fit Index (IFI) = 0.984, and Root Mean Square Error of Approximation (RMSEA) = 0.030 (see Table 5), confirming the model’s suitability for subsequent path analysis.

**Table 5:** The goodness of fit indices for the measurement model and research model.

Model	x^2^	x^2^/df	TLI	CFI	IFI	RMSEA
Measurement model	571.050	1.407	0.981	0.984	0.984	0.030
Research model	1033.007	2.425	0.934	0.939	0.940	0.054
Recommended criteria	*p* > 0.05	<5.0	>0.90	>0.90	>0.90	<0.08

### 4.2 Structural model

The proposed research model was examined using SEM, and the results demonstrated an acceptable model fit (χ^2^/df = 2.425, TLI = 0.934, CFI = 0.939, IFI = 0.940, RMSEA = 0.054; see Table 5). All eight hypotheses proposed in this study were empirically supported by the data (see Table 6).

**Table 6:** The hypothesis test results.

Hypotheses	Hypothesized path	B	β	S.E	t	Result
H1	NA → FE	0.366	0.343	0.049	7.501***	Supported
H2	AM→FE	0.245	0.215	0.052	4.749***	Supported
H3	HO → FE	0.223	0.180	0.056	4.005***	Supported
H4	FA → FE	0.445	0.404	0.051	8.699***	Supported
H5	AP → FE	0.111	0.121	0.041	2.700**	Supported
H6	FE → ME	0.470	0.515	0.052	9.047***	Supported
H7	ME→RI	0.421	0.356	0.073	5.795***	Supported
H8	FE → RI	0.321	0.297	0.063	5.126***	Supported

NA, nature; AM, ambient; HO, hospitality; FA, farming activities; AP, agricultural products; FE, flow experience; ME, memory; RI: revisit intention. *** *p* < 0.001, ** *p* < 0.01.

The path analysis results showed that all five dimensions of the rural tourism experience had significant positive effects on flow experience: natural environment (*β* = 0.366, *t* = 7.501, *p* < 0.001), atmosphere (*β* = 0.245, *t* = 4.749, *p* < 0.001), hospitality culture (*β* = 0.223, *t* = 4.005, *p* < 0.001), participatory activities (*β* = 0.445, *t* = 8.699, *p* < 0.001), and creative agricultural products (*β* = 0.111, *t* = 2.700, *p* < 0.01). Thus, hypotheses H1 through H5 were all supported. These findings suggest that diverse and engaging rural tourism settings effectively enhance visitors’ immersive experiences.

**Figure 2:**
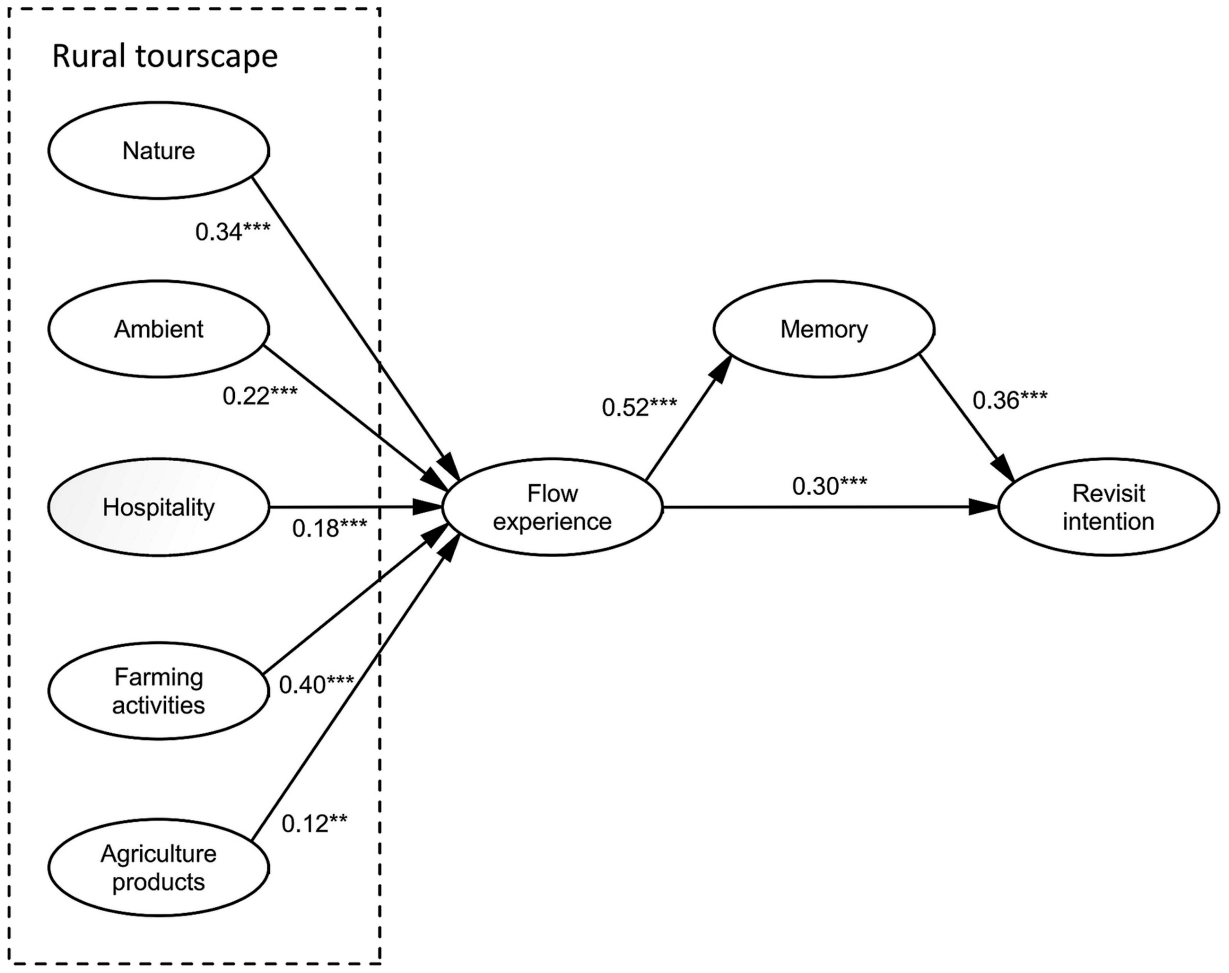
Structural model. *** significant at *p* < 0.001, ** significant at *p* < 0.01.

In terms of the path mechanism, flow experience had a significant positive effect on memory (*β* = 0.470, *t* = 9.047, *p* < 0.001), thereby supporting H6. Memory, in turn, had a significant positive effect on revisit intention (*β* = 0.421, *t* = 5.795, *p* < 0.001), providing empirical support for H7. Additionally, flow experience exerted a significant direct effect on revisit intention (*β* = 0.321, *t* = 5.126, *p* < 0.001), confirming H8. These results indicate that immersive experiences in rural tourism not only enhance memory depth but also, directly and indirectly, through memory, promote visitors’ revisit intention. The validated structural model is illustrated in Figure 2.

### 4.3 Mediation and serial mediation effects

As shown in Table 7, flow experience (FE) served as a significant mediator in the relationships between each independent variable—natural environment (NA), atmosphere (AM), hospitality culture (HO), participatory activities (FA), and agricultural products (AP)—and revisit intention (RI), thereby supporting the proposed hypotheses. Among these, the strongest mediating effect was observed in the FA → FE → RI pathway (*β* = 0.120), while the weakest was found in the AP → FE → RI pathway (*β* = 0.036). These findings underscore the critical role of FE in linking diverse aspects of the rural tourscape to revisit intention.

**Table 7:** Mediation analysis.

Parameter	Estimate	SE	Lower	Upper	P
NA-FE-RI	0.102	0.031	0.051	0.173	0.000
AM-FE-RI	0.064	0.020	0.032	0.113	0.000
HO-FE-RI	0.053	0.021	0.021	0.106	0.001
FA-FE-RI	0.120	0.035	0.062	0.202	0.000
AP-FE-RI	0.036	0.016	0.011	0.076	0.003
NA-FE-ME	0.177	0.037	0.108	0.259	0.000
AM-FE-ME	0.111	0.031	0.052	0.175	0.000
HO-FE-ME	0.092	0.033	0.034	0.163	0.002
FA-FE-ME	0.208	0.040	0.135	0.291	0.000
AP-FE-ME	0.062	0.024	0.019	0.115	0.005
NA-FE-ME-RI	0.063	0.019	0.033	0.109	0.000
AM-FE-ME-RI	0.039	0.015	0.017	0.076	0.000
HO-FE-ME-RI	0.033	0.014	0.012	0.067	0.001
FA-FE-ME-RI	0.074	0.021	0.039	0.125	0.000
AP-FE-ME-RI	0.022	0.010	0.007	0.049	0.003

NA, nature; AM, ambient; HO, hospitality; FA, farming activities; AP, agricultural products; FE, flow experience; ME, memory; RI, revisit intention.

Moreover, memory (ME) also exhibited significant mediating effects in the relationships between the same set of independent variables (NA, AM, HO, FA, AP) and revisit intention (RI), providing further support for the corresponding hypotheses. The most substantial mediation occurred in the FA → FE → ME pathway (*β* = 0.208), whereas the AP → FE → ME pathway showed the weakest effect (β = 0.062). All bias-corrected confidence intervals excluded zero (p < 0.01), confirming the robustness of ME’s mediating role.

Moreover, the chain mediation analysis showed that FE and ME had a significant joint effect as serial mediators in the relationships between each of the independent variables and RI. All bias-corrected confidence intervals for these serial mediation paths excluded zero (p < 0.01), indicating strong and reliable chain mediation effects. Overall, the findings provide robust evidence that flow experience and memory jointly mediate the positive influence of rural tourscapes on tourists’ revisit intention.

### 4.4 fsQCA results

#### 4.4.1 Calibration of variables

Before performing fsQCA, all latent variables, which had been used in SEM, were transformed into observable composite indicators. Following established fsQCA practices (Ragin, 2009; Fiss, 2011), the measurement items under each latent construct were aggregated using the additive approach, which is widely adopted in fsQCA research due to its simplicity, transparency, and minimal information loss (Misangyi et al., 2017).

After generating composite indicators, all variables were calibrated into fuzzy-set membership scores using the direct method. The 95th, 50th, and 5th percentiles of each variable were used as the anchors for full membership (1), the crossover point (0.5), and full non-membership (0), respectively (Ragin, 2009; Schneider and Wagemann, 2012). To avoid membership scores falling exactly at the crossover point, each value was adjusted by adding 0.001 to reduce ambiguity in set assignment (Fiss, 2011). The calibration anchors are presented in Table 8.

**Table 8:** Calibration anchors for fuzzy-set membership scores.

Percentiles	NA	AM	HO	FA	AP	FE	ME	RI
5	6.20	4.20	8.00	8.00	4.00	5.00	6.00	4.00
50	17.00	12.00	12.00	16.00	12.00	16.00	11.00	16.00
95	23.80	16.00	18.00	20.00	15.00	20.00	15.00	20.00

NA, nature; AM, ambient; HO, hospitality; FA, farming activities; AP, agricultural products; FE, flow experience; ME, memory; RI, revisit intention.

#### 4.4.2 Analysis of necessary conditions

Table 9 presents the results of the necessity analysis for high revisit intention (RI). A condition is considered necessary when its consistency is ≥ 0.90 (Ragin, 2009), with coverage ≥ 0.60 as a complementary criterion to avoid Type I errors (Mattke et al., 2021).

**Table 9:** Analysis of necessary conditions for high RI.

Conditions	Consistency	Coverage
NA	0.734	0.727
~NA	0.638	0.672
AM	0.795	0.656
~AM	0.522	0.699
HO	0.796	0.695
~HO	0.556	0.684
FA	0.741	0.739
~FA	0.651	0.681
FE	0.772	0.778
~FE	0.572	0.593
ME	0.784	0.753
~ME	0.548	0.598

NA, nature; AM, ambient; HO, hospitality; FA, farming activities; AP, agricultural products; FE, flow experience; ME, memory; RI, revisit intention. ~ indicates the absence of the condition.

Across all antecedent conditions and their negations, consistency scores ranged from 0.522 to 0.796, which fall below the 0.90 threshold. Although coverage levels were moderate (0.593–0.778), no condition, either its presence or absence, can be considered necessary for producing high RI.

These results indicate that revisit intention does not rely on any single antecedent condition but instead demonstrates configurational causality, warranting further sufficiency analysis.

#### 4.4.3 Sufficiency analysis results

In the sufficiency analysis, the truth table was constructed using a consistency threshold of 0.80 and a PRI threshold of 0.65 (Fiss, 2011; Schneider and Wagemann, 2012). To avoid spurious low-frequency configurations, a frequency cutoff of seven cases (1.5% of the sample) was applied. After logical minimization using the Quine–McCluskey algorithm, eight sufficient configurations leading to high revisit intention (RI) were obtained (Table 10). The overall solution coverage is 0.660, and the solution consistency is 0.838, indicating strong explanatory adequacy of the configurational model.

**Table 10:** Configurations leading to high RI.

Conditions	Config. 1	Config. 2	Config. 3	Config. 4	Config. 5	Config. 6	Config. 7	Config. 8
NA		●		●	ⓧ	ⓧ	●	●
AM	●	●	●	●	●	●	●	
HO	●	●	●	●	●	●	●	●
FA	●	●	●					●
AP			●	ⓧ	●	ⓧ	●	●
FE		●	●	ⓧ	ⓧ	●	●	●
ME	●			●	●	●	●	●
Raw coverage	0.565	0.550	0.503	0.308	0.329	0.323	0.469	0.464
Unique coverage	0.007	0.014	0.006	0.003	0.009	0.003	0.009	0.013
Consistency	0.882	0.900	0.888	0.915	0.918	0.919	0.916	0.921
Overall consistency	0.838							
Overall coverage	0.660							

NA, nature; AM, ambient; HO, hospitality; FA, farming activities; AP, agricultural products; FE, flow experience; ME, memory; RI, revisit intention. ● and ● indicates the presence of the condition; ⓧ indicates the absence of the condition; blank cells denote that both the presence and absence of the condition may appear within the configuration; ● and ⓧ represent peripheral (non-core) conditions.

To move beyond general patterns, several representative configurations were extracted to highlight the dominant causal mechanisms. The first pathway (C1) is characterized by farming activities (FA) and memory (ME) as core conditions, complemented by ambient atmosphere and hospitality. This configuration indicates that embodied, hands-on participation coupled with strong episodic memory provides a powerful causal route to high RI, emphasizing the role of experiential engagement in shaping post-visit behavioral tendencies.

The second representative channel (C2) has joint core drivers such as flow experience (FE) and agricultural products (AP). This mechanism reflects an immersion-based process in which attentional absorption and engagement with local products enhance revisit intention. The third pathway (C3) involves nature (NA) as a core antecedent, memory (ME), and agricultural products (AP). This arrangement suggests that natural exposure provides psychological restoration. Memory and product experience further support cognitive and emotional resonance, jointly enhancing high RI.

Across all eight configurations, flow experience (FE) and memory (ME) frequently emerge as core conditions, underscoring their central roles in driving revisit intention. FA, AP, and NA function as core conditions in different configurations, revealing heterogeneous yet convergent causal mechanisms. By contrast, ambient atmosphere (AM) and hospitality (HO) mainly appear as peripheral elements that strengthen but do not independently determine the causal pathways. Overall, the results demonstrate clear equifinality: high revisit intention can be achieved via several qualitatively distinct configurations. No single condition is sufficient on its own; instead, different combinations of rural tourscape attributes, flow experience, and memory constitute sufficient causal packages leading to high revisit intention.

## 5 Discussion and implications

By applying CAT as a theoretical background, the present paper develops and confirms a path model of Rural Tourscape–Flow Experience–Memory–Revisit Intention. This model empirically reveals the internal relationships between tourists’ subjective perceptions and their emotional experiences. These findings indicate that tourists who consider such elements as rural natural settings, social atmosphere, and cultural attributes to be goal-congruent and manageable tend to experience immersive flow states and create positive memories more frequently. These emotional reactions, in turn, strengthen emotional bonds to the destination and improve revisit intentions. The results not only confirm the proposed model but also extend the argument of Ding and Hung (2021), demonstrating that subjective evaluation plays a decisive role in shaping emotional experiences and subsequent behavioral intentions. Notably, this paper shows that tourists are engaged in cognitive processes rather than passively receiving environmental stimuli. Tourists actively construct the meaning of tourism contexts through cultural identification, situational adaptation, and social interaction. This renders the relationships among perception, emotion, and behavior more dynamic and interactive. Besides the symmetric and net-effect findings, the results of the fsQCA also show that there are numerous conjunctural pathways to high revisit intention, meaning that no one particular dimension of the tourscape is required and that various combinations of environmental and experiential circumstances lead to revisiting behavior.

The paper also explored how the structural dimensions of the rural tourscape influence the creation of flow experience, hence determining the emotional motivators of revisit intention. The findings reveal that the five dimensions are all significant and positive predictors of flow, and natural environments and participatory activities have the highest path coefficients. This occurs because these two dimensions enhance psychological involvement and promote intensive sensory interaction. Natural environments provide visual gratification and a relaxed experience that allows tourists to focus and experience psychological renewal, which are positive circumstances for entering a flow state. Participatory farming activities provide clear goals, immediate feedback, and hands-on cultural involvement, allowing tourists to feel competent, focused, and closely connected to local life. These experiential aspects increase engagement, realism, and emotional appeal that lead to greater immersion and consequently more impact on flow than the other dimensions of tourscape. This means that the experience of rural tourism is mostly induced by the aesthetic orientation of natural environments and the practical experiences of hands-on activities. As has already been established, natural settings are important in terms of immersion as they provoke fascination and a transcendent emotional state, which reinforces satisfaction and place attachment (Chen et al., 2024; Huang et al., 2022). Furthermore, they evoke positive emotions and form memories due to their capacity to stimulate the human organism softly (Johnsen and Rydstedt, 2013).

The clarity of goals and feedback in farming activities also contributes to the increase in tourists’ feelings of control and competence and allows them to reach a flow state. Immersion is usually perceived when experiences become both challenging and demanding, as was pointed out by Ding and Hung (2021) in the issue of festival tourism. Similarly, the emotional and social atmosphere, alongside the hospitality of the local inhabitants, creates a sense of place identity and social attachment, enhancing tourists’ emotional bonds with the destination. Such emotional attachments provide the basis for valuable memory formation and, eventually, the reinforcement of revisit intention. Agricultural products that are unique but creative are also tangible bearers of local culture. Their local and unique features not only satisfy the needs of tourists for a new and authentic experience but also offer sensory and symbolic stimuli, which enhance immersion and enrich the cultural aspect of flow (Zhang et al., 2022; Zhang and Huang, 2025).

Further discussion showed that flow experience has a dual mediating effect between the rural tourscape and revisit intention: indirect, through memory, and direct, through influencing behavioral tendencies. On the one hand, flow experience as an altered state of strong concentration and enjoyment makes situational experiences vividly encoded and permanently stored. Positive memories, on the other hand, are reusable in future decisions, which enhance positive cognitive and emotional appraisals of the destination. This observation aligns with tourism psychology’s proposition of an emotion–memory–behavior chain mechanism. As Huang and Bu (2022) argue, the immediate experience at the destination not only influences behavioral intention, but is also influenced by the intensity of emotions encoded in memory and reactivated during recall. The reactivation of emotional memory has been demonstrated as a primary psychological mechanism driving revisit motivation. This is particularly evident in rural tourism settings, where tourists seek both an escape from everyday life and a sense of cultural belonging (Marks-Tarlow and Panksepp, 2015).

### 5.1 Theoretical implications

This study extends CAT to rural tourism by applying it beyond its traditional use in coping with stress and emotion regulation (Choi and Choi, 2019; Tan et al., 2023; Meng and Luo, 2024). Although the S-O-R framework can effectively explain the relationships among stimulus, organism, and response, it is limited in revealing the role of tourists’ cognitive appraisal processes in shaping emotions and behavioral intentions (Zhu et al., 2025a,b). By incorporating primary and secondary appraisals, this study clarifies how tourists evaluate goal congruence and perceived controllability within rural tourscape settings. CAT is able to capture the complexity of tourists’ cognitive and emotional processes, which the S-O-R framework cannot adequately capture (Lee et al., 2026).

This study highlights the central role of cognitive appraisal in the formation of tourists’ immersive emotions. The development of flow experiences and positive memories is influenced not only by environmental stimuli but also by tourists’ evaluations of the relevance of the experience and their own agency (Li et al., 2026). By developing the Rural Tourscape–Flow Experience–Positive Memory–Revisit Intention pathway model, the proposed research develops a theoretically grounded interpretation of the connection between environmental perceptions and behavioral intentions transformed into sustained behavior. Combining flow-based immersion and memory-based psychological persistence shows a dual-process mechanism that explains how temporary immersion states develop into enduring emotional perceptions that, in turn, direct decision-making. This incorporates the structured cognitive-emotional process that augments theoretical knowledge on sustainable tourist behavior.

In addition to linear and symmetric results presented by SEM, the results achieved by fsQCA reveal that revisit intention may be caused by various conjunctural groupings of rural tourscape factors and experience circumstances. It brings about causal asymmetry and equifinality to the field of rural tourism research, indicating that no single factor or sequential process can be relied upon. Rather, different experiential setups can all induce high revisit intention. This configurational approach is an addition to mechanism-based theories of tourist behavior like CAT because it gives an overall interaction of both environmental and psychological influences, hence enhancing explanations of tourist behavior theories.

This study empirically demonstrates that the influence of rural environmental characteristics on tourist behavior arises not merely from their objective features, but through tourists’ subjective cognition and emotional processing, as suggested by Liu and Lin (2024) in the five-dimensional rural tourscape model. The results support the conceptual assumption that perception into action conversion is mediated by means of the experiential environment, and they contextualize the tourscape construct to a rural cultural environment. This will help to refine the theory of tourscape and will offer an empirical basis for conducting future research on sustainable tourist behavior across cultures and regions.

### 5.2 Practical implications

According to CAT, the study establishes how tourists’ subjective perceptions of rural tourscape influence their revisit intention through the dual mediating effects of flow experience and emotional memory. Destination management and product design may be guided by these findings.

First of all, aesthetically speaking, as far as spatial and environmental design is concerned, the nature dimension contributes significantly to the process of flow experiences and favorable memories. The destinations ought to be aimed at making healthy designs of restorative landscapes with proper visual exposure, environmental texture, and walkability, and ensuring that any adverse effects of noise, light, and crowding are eliminated. Low-interference, aesthetically pleasing natural settings, such as forest trails, waterfront boardwalks, and countryside camping areas, facilitate tourists’ immersion and create positive, lasting first impressions that enhance revisit intentions (Zhong et al., 2024). The use of local ecological aesthetics in materials and spatial language may also enhance place-based memory and emotional recognition (Shao et al., 2024). The use of sensory-based landscape features, including phenomenal sounds, tangible materials, and unique smells, can complement a multisensory experience and contribute to further immersion and tourists’ flow experience (Zhai et al., 2023).

Second, in socio-cultural terms, such aspects as atmosphere and hospitality culture positively increase the degree of involvement and emotional recognition by tourists. Destination managers should promote local involvement and cultural synergy by organizing activities with community participation, such as guided tours and welcome ceremonies, to foster a friendly and inclusive atmosphere. Service training and behavioral guidance can be used to strengthen hospitality quality. Interactive products can be extended further through interactive programs, including Evening Talks in the Village or Shared Meals with Locals, and this will help enhance the feeling of social belonging in tourists and thus spread motivation towards returning (Folgado-Fernández et al., 2021).

Third, in terms of activity planning and product development, the size of participatory activities and creative agricultural products has been found to be effective and to trigger flow experience, along with strengthening emotional bonding by activating memories (Zhang et al., 2022). Tourism experiences should emphasize hands-on activities and cultural richness, such as seasonal farming, intangible cultural heritage workshops, and narrative-based rural markets. Incorporation of emotional symbols, local stories, and ritual provisions is useful for eliciting rich and memorable memories (Sterchele, 2020). Further, as part of the idea of green souvenirs, destinations can create locally unique and greener items that become cultural indicators, thereby making the emotional impacts create longer-lasting results and repeat-visit behavior (Zhang and Huang, 2025). It will be possible to design participatory farming workshops or storytelling experiences and enhance hands-on activity, cultural resonance, and the possibility of entering a flow state.

Moreover, in order to strengthen the memory-based emotional bond and continuity of behavior, strategic memory activation nodes are supposed to be placed along important tourist paths. The design objects can consist of viewpoints, cultural monuments, or interactive spaces with the assistance of digital guides, interpretation panels, or AR-based narratives to provoke more attention and record behavior. To market their destinations, marketers have to work better on content strategy in order to capitalize on the exchange of short clips, visitor accounts, and user-generated content (UGC) based on such topics as slowed-down lives, country recollection, and emotional experiences. This will also assist in strengthening the frequency and emotional intensity of recalled memories, thereby enhancing revisit intention (Wu and Peng, 2025). Embedding cultural rituals or simple AR-based memory triggers can further strengthen emotional recall and reinforce tourists’ long-term connection with the destination.

Lastly, the fsQCA findings represent a significant managerial implication that high revisit intention could be realized with more than one alternative setup instead of one universal solution. Such a result implies that destinations do not have to be better when compared to all dimensions of tourscape at the same time. Rather, managers can maximize development by reinforcing particular combinations that best fit with local resources. In particular, one could consider matching natural settings with the involvement of these experiences, merging social tone with innovative product lines, or exploiting the culture of strong hospitality along with recall. This perspective supports differentiated and context-sensitive development strategies, enabling rural destinations to pursue varied yet equally effective paths to enhancing revisit intentions while optimizing resource allocation.

## 6 Limitations and future research directions

This research has a number of limitations, indicating future research directions. To begin with, the sample’s geographical scope restricts the generalizability of the results. The sample was chosen from some of the coastal rural tourism destinations. Even though these sites represent the common landscape structure and type of tourism, they do not entirely represent the variety of rural environments encountered in mountainous landscapes, lake landscapes, or inland western China. Conducting the study in a coastal environment may limit the external validity of the proposed model. Tourists’ cognitive appraisals, emotional reactions, and behavioral intentions are likely to differ in inland, mountainous, or culturally diverse rural settings. The model needs to be proven through cross-regional or cross-cultural comparisons in future studies to determine its application outside the study. This study employed anonymous responses, adjusted the scale endpoints for independent and dependent variables, and randomized the item order. Future research could further reduce potential common-method bias by collecting data at different times or from multiple sources.

Second, a quantitative method was used in the study as a primary approach to test the hypothesized model. Although SEM can be used to understand changes in behavior based on their causal relationships, it offers little understanding of the processes involved in the flow of memory, which accentuates behavioral intentions. Furthermore, as much as fsQCA helps in complementing SEM in detecting multiple satisfactory configurations, the process still uses calibration thresholds and composite indicator constructions, which can affect the results of the configuration. Future studies can involve using other types of calibration anchors, checking robustness, or using multi-method fsQCA to enhance reliability. To represent psychological and emotional processes of tourists in a more all-encompassing way, it is possible to include in-depth interviews, travel diaries, psychological tracking, or even physiological measurements.

Third, the model does not incorporate moderating variables, potentially overlooking the effects of personal or situational factors. Relationships between flow, memory, and revisit intention could be moderated by variables like environmental awareness, personality characteristics (e.g., emotional stability and openness), travel motivation, and frequent visits. The inclusion of such moderators in future research would contribute to an improved explanatory power and flexibility of this model. Lastly, this research addresses revisit intention as the key behavioral outcome, and other related extended responses like loyalty, word-of-mouth communication, or destination brand identification are not exhaustively studied in it. Future research may adopt a multidimensional perspective on tourist behavioral outcomes to expand the applicability of the “experience-memory-behavior” framework and explore how immersive experiences foster long-term emotional attachment and destination loyalty.

## Data Availability

The original contributions presented in the study are included in the article/Supplementary material, further inquiries can be directed to the corresponding author/s.
